# Antineutrophil cytoplasmic antibody mediated glomerulonephritis associated with levamisole-adulterated cocaine

**DOI:** 10.5414/CNCS108385

**Published:** 2014-12-15

**Authors:** Zita Shiue, Bairbre McNicholas, Fionnuala Cormack, Shreeram Akilesh

**Affiliations:** 1 Division of Nephrology and; 2 Department of Pathology, University of Washington, Seattle, WA, USA

**Keywords:** ANCA, glomerulonephritis, cocaine, levamisole

## Abstract

Levamisole-adulterated cocaine has increased in prevalence over the last decade and is known to be associated with antineutrophil cytoplasmic antibodies (ANCA). Dermatologic manifestations of levamisole exposure, including cutaneous vasculitis, are widely appreciated; less is known about its effects on the kidney. We report two cases of patients with a history of cocaine abuse and levamisole-induced cutaneous vasculitis, who developed acute kidney injury in the setting of elevated ANCA titers. Renal biopsies of both revealed pauci-immune complex glomerulonephritis with diffuse crescentic disease. These cases demonstrate a rare but serious complication of long-term cocaine use.

## Introduction 

Levamisole was first discovered as an adulterant in 2003 and is now thought to be present in more than 70% of all cocaine samples [1]. Increased use of cocaine containing levamisole has led to a number of complications, including agranulocytosis, cutaneous vasculitis, and necrosis [2, 3, 4, 5]. Serologically, levamisole exposure is associated with antineutrophil cytoplasmic antibodies (ANCA) and occasionally anti-double stranded DNA and lupus anticoagulant [6]. Although dermatologic manifestations are well-described, there are only a few reported cases of focal crescentic glomerulonephritis associated with cocaine and levamisole exposure [7, 8]. Here, we present two cases of diffuse crescentic glomerulonephritis associated with long term cocaine use, likely induced by levamisole-adulterated cocaine. 

## Case 1 

**Figure 1. Figure1:**
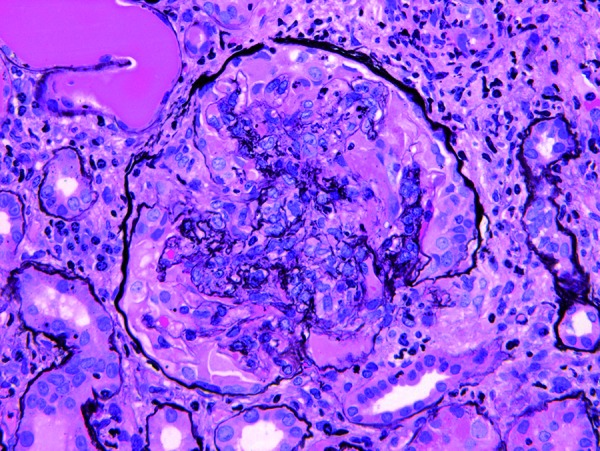
Representative glomerulus from case 1. There is prominent segmental necrosis with rupture of glomerular basement membranes and formation of a circumferential cellular crescent. The surrounding tubulointerstitium is inflamed (Jones methanamine silver, 400× original magnification).

A 34-year-old female, with a history of cocaine use, presented with a creatinine of 4.1 mg/dL during an admission for cellulitis. One year prior, she was admitted for polyarthralgias and an auricular rash. Then, her ANCA titer was 1 : 65,536, with positive antimyeloperoxidase (MPO) antibodies of 5.8 (0.0 – 0.7) and anti-proteinase-3 (PR3) antibodies of 5.2 (0.0 – 0.7). Her creatinine was 0.5 mg/dL, and urinalysis (UA) revealed hematuria. Antinuclear antibody (ANA) titers were 1 : 80. Rheumatoid factor (RF) and cryoglobulins were negative. She had a positive hepatitis C antibody but negative polymerase chain reaction (PCR), and her hepatitis B antibody was nonreactive. Given her long history of cocaine use, she was diagnosed with levamisole-induced cutaneous vasculitis.[Fig Figure1]

At this presentation, she complained of lower extremity edema, joint pain, and hematuria. She denied fever, rash, oral ulcers, sore throat. Her exam was significant for systolic blood pressures of 130 – 150s mmHg, a known 2/6 systolic murmur, 2+ edema, and no rash. Labs revealed a creatinine of 4.1 mg/dL, potassium 4.8 mEq/L, white blood cell count (WBC) 8,410 per µL, hemoglobin 6.8 g/dL, and platelets of 482,000 per µL. ANCA titers were 1 : 8192, MPO-ANCA > 6, and PR3-ANCA of 1. ANA, hepatitis C RNA, and cryoglobulins were negative. HIV was nonreactive. C3 and C4 levels were normal. Chest X-ray was normal. A urine protein-to-creatinine ratio (PCR) was 2.6 g/g and urine microscopy revealed red cell casts. She initially refused biopsy and did not return to the clinic. 

She presented 3 weeks later with a creatinine of 4.0 mg/dL. A biopsy revealed diffuse necrotizing and crescentic glomerulonephritis, with active and chronic features. Approximately 3 – 5 glomeruli per level section (of 9 – 11) exhibited segmental scarring or sclerosis with segmental obliteration of the glomerular tufts by matrix and occupation of the adjacent urinary spaces by fibrous to fibrocellular crescents. The cortical parenchyma revealed diffuse interstitial fibrosis and tubular atrophy. Immunofluorescence and electron microscopy did not show immune deposits, consistent with a pauci-immune complex etiology. Although a toxicology was not obtained, the pathology was felt to be most consistent with levamisole-induced glomerulonephritis given her previous history of cutaneous vasculitis. The patient could not be reached to discuss the results. She presented one week later with a serum creatinine of 6.4 mg/dL and a potassium of 7.5 mEq/L, requiring urgent dialysis. She was treated with plasmapheresis and cyclophosphamide. However, she remained dialysis-dependent. She died 4 months later due to unrelated trauma.

## Case 2 

**Figure 2. Figure2:**
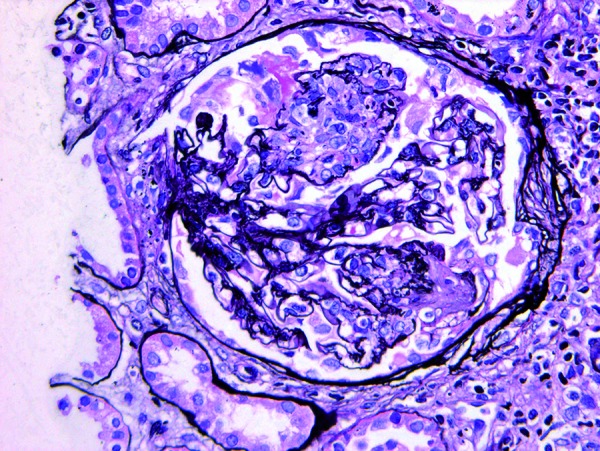
Representative glomerulus from case 2. There is segmental endocapillary hypercellularity with rupture of the glomerular basement membrane and formation of highly eosinophilic fibrinous exudate. (Jones methenamine silver, 400× original magnification).

A 53-year-old Caucasian male with a long history of cocaine use presented with a foot abscess and a serum creatinine of 7.9 mg/dL, an increase from 2.0 mg/dL 4 months prior. Notably, he was hospitalized 2 years prior for painful purpuric patches and hemorrhagic vesicles over his abdomen and lower extremities. At the time, he had a creatinine of 1.4 mg/dL, C- reactive protein 19.8 mg/L, RF 23 IU/mL (normal < 13), negative ANA, positive cryoglobulins at 3 days (1.4%), C3 71 mg/dL (87 – 247), C4 5 mg/dL (10 – 37). Hepatitis C antibody was positive, with a viral load of 630,000 IU/mL. ANCA titers were 1 : 4,096, and an MPO-ANCA level was 4.6. His urine toxicology was positive for cocaine, and his rash was felt to be most consistent with levamisole-induced vasculitis. Urinalysis was not performed. Creatinine was 1.1 mg/dL at discharge.[Fig Figure2]

On this admission, laboratory tests also revealed a WBC of 4,400 per µL and hemoglobin of 7.4 g/dL. Urinalysis showed 3+ blood and 3+ protein, and microscopy revealed rare dysmorphic red cells. His MPO-ANCA level was now > 80. A chest X-ray was negative, and he had no sinus pain or hemoptysis. His kidneys appeared normal on ultrasound. A renal biopsy revealed diffuse crescentic glomerulonephritis, ~ 40 – 100% of the glomeruli showed either cellular or fibrocellular crescents, some of which also demonstrated glomerular basement membrane breakage and focal fibrin deposition in the capillary tuft of Bowman’s space. The tubular parenchyma also demonstrated mild to moderate interstitial fibrosis and tubular atrophy. There was no obvious vasculitis identified. There was no evidence of immune complex mediated disease by immunofluorescence or electron microscopy. Given his history of cutaneous vasculitis, this was thought to be related to levamisole-adulterated cocaine. He was treated with intravenous methylprednisone, rituximab, and 7 sessions of plasmapheresis. Creatinine improved to 5.4 mg/dL at discharge. However, he re-presented 1 week later with hyperkalemia and volume overload, and dialysis was initiated. Presently, he remains dialysis-dependent. 

## Discussion 

The use of levamisole as an adulterant has increased considerably over the last 10 years, with its prevalence reaching 73.2% of all cocaine samples in 2009 [1]. Levamisole is a broad spectrum nicotinic antihelminthic with immunomodulatory properties [9], initially used to treat rheumatoid arthritis, nephrotic syndrome, and colon cancer. However, it was withdrawn from the market in 2000 due to its association with agranulocytosis, and it is now primarily used in veterinary medicine [6]. Levamisole’s immunomodulatory effect is thought to be caused by its ability to increase macrophage chemotaxis and T-cell function, reduce suppressor T-cell function, induce granulocyte maturation, enhance dendritic cell maturation, and up-regulate toll-like receptors [6, 10, 11]. 

Complications from levamisole, including cutaneous vasculitis and agranulocytosis, were initially described in the 1970s [12, 13]. Later, cutaneous vasculitis was described with concomitant ANCA positivity in children undergoing treatment for nephrotic syndrome [14, 15, 16, 17]. Lesions were generally confined to the ears, cheeks, lower limbs, and associated with arthralgias. With increasing levamisole exposure amongst cocaine users, additional adverse effects have been reported, including bullae disorders, pulmonary hemorrhage, hyponatremia, and granulomatosis [6, 18]. Patients with levamisole-induced vasculitis typically have a history of long-standing cocaine use, and their manifestations are thought to be distinct from cocaine use alone. Cocaine-induced vasculitis usually presents with vasoconstrictive features, such as cerebral vasculitis, digital necrosis, and gangrene [3, 7]. Earlobe lesions are not typical, and ANCA tends to be negative except in cases of cocaine-induced midline destructive lesions (CIMDL) [2,19]. Levamisole itself can be measured in the urine, but is usually difficult to detect due to its short half-life of 5.6 hours [20]. 

How levamisole exposure results in features consistent with ANCA vasculitis is not entirely clear. Drug-induced vasculitis is typically associated with antibodies to MPO-ANCA or PR3-ANCA, but antibodies to other components of neutrophil granules, including human neutrophil elastase (HNE) and lactoferrin, have also been reported [2]. ANCA can induce neutrophil activation upon engagement of MPO-ANCA and PR3-ANCA [21] as well as HNE. In a study of cocaine users, HNE-ANCA was identified in up to 80% of cases [22]. HNE and PR3 are from the same chymotrypsin family of serine proteases, but induce formation of distinct antibodies. In one series, antibodies to HNE were found in 100% of patients with levamisole induced vasculitis [23]. HNE antibodies are rarely found in nondrug-related vasculitis and may be a marker for disease. 

Despite a growing appreciation of cutaneous vasculitis, there has only been one case series, to our knowledge, describing renal manifestations attributed to levamisole [7]. This series reviewed 30 cases of ANCA positivity associated with cocaine ingestion. All had positive MPO antibodies, and 50% also had positive PR3 antibodies. Two patients developed severe acute kidney injury with concurrent UAs that were positive for blood and protein. A biopsy of one of the patients revealed pauci-immune focal necrotizing crescentic glomerulonephritis. Despite immunosuppressive treatment, both had residual chronic kidney disease (estimated glomerular filtration rate (GFR) < 30 mL/min per 1.73 m^2^) but did not require dialysis. 

Although we do not have documented levamisole positivity in our subjects, both had previous presentations with cutaneous vasculitis and ANCA positivity that are highly suspicious for levamisole exposure. Of note, their dermatologic manifestations preceded their renal disease by at least 1 – 2 years. We speculate that pauci-immune complex glomerulonephritis represents a late but serious complication from years of levamisole exposure, and routine evaluation of renal function and urinalysis should be performed in these patients. In contrast to previously reported cases, our patients presented with much more aggressive diffuse crescentic glomerulonephritis. Levamisole-associated cutaneous necrosis has improved with plasmapheresis in some cases [24], but outcomes in renal failure are unknown. Despite treatment with plasmapheresis, high-dose steroids, and a cytotoxic agent, both our patients were dialysis-dependent. 

## Conclusion 

Over the last decade, there has been a wider appreciation of levamisole-induced vasculitis. While dermatologic manifestations are well described, less is known about levamisole’s effect on the kidney. We report two diffuse crescentic glomerulonephritis, a rare but serious complication of long-term use of cocaine adulterated with levamisole. Therefore, a careful drug use history should be obtained in all patients with ANCA-positive glomerulonephritis. Treatment remains a challenge given the paucity of data and its presence in a population with poor socioeconomic support. 

## Conflict of interest 

No conflicts of interest to disclose. No support or financial disclosures to acknowledge. 

## References

[1] Buchanan JA Heard K Burbach C Wilson ML Dart R Prevalence of levamisole in urine toxicology screens positive for cocaine in an inner-city hospital. JAMA. 2011; 305: 1657–1658. 21521846 10.1001/jama.2011.531

[2] Graf J Lynch K Yeh CL Tarter L Richman N Nguyen T Kral A Dominy S Imboden J Purpura, cutaneous necrosis, and antineutrophil cytoplasmic antibodies associated with levamisole-adulterated cocaine. Arthritis Rheum. 2011; 63: 3998–4001. 22127712 10.1002/art.30590

[3] Khan TA Cuchacovich R Espinoza LR Lata S Patel NJ Garcia-Valladares I Salassi MM Sanders CV Vasculopathy, hematological, and immune abnormalities associated with levamisole-contaminated cocaine use. Semin Arthritis Rheum. 2011; 41: 445–454. 22152487 10.1016/j.semarthrit.2011.04.010

[4] Poon SH Baliog CR Sams RN Robinson-Bostom L Telang GH Reginato AM Syndrome of cocaine-levamisole-induced cutaneous vasculitis and immune-mediated leukopenia. Semin Arthritis Rheum. 2011; 41: 434–444. 21868067 10.1016/j.semarthrit.2011.05.009

[5] Morris GW Mason BC Harris Sprunger R Hake Harris H White LA Patterson DA Levamisole-adulterated cocaine: a case series. J Am Board Fam Med. 2012; 25: 531–535. 22773722 10.3122/jabfm.2012.04.110287

[6] Lee KC Ladizinski B Federman DG Complications associated with use of levamisole-contaminated cocaine: an emerging public health challenge. Mayo Clin Proc. 2012; 87: 581–586. 22677078 10.1016/j.mayocp.2012.03.010 PMC3498128

[7] McGrath MM Isakova T Rennke HG Mottola AM Laliberte KA Niles JL Contaminated cocaine and antineutrophil cytoplasmic antibody-associated disease. Clin J Am Soc Nephrol. 2011; 6: 2799–2805. 21980179 10.2215/CJN.03440411 PMC3255368

[8] Neynaber S Mistry-Burchardi N Rust C Samtleben W Burgdorf WH Seitz MA Messer G Wollenberg A PR3-ANCA-positive necrotizing multi-organ vasculitis following cocaine abuse. Acta Derm Venereol. 2008; 88: 594–596. 19002345 10.2340/00015555-0514

[9] Sajid MS Iqbal Z Muhammad G Iqbal MU Immunomodulatory effect of various anti-parasitics: a review. Parasitology. 2006; 132: 301–313. 16332285 10.1017/S0031182005009108

[10] Renoux G Renoux M Teller MN McMahon S Guillaumin JM Potentiation of T-cell mediated immunity by levamisole. Clin Exp Immunol. 1976; 25: 288–296. 782749 PMC1541355

[11] Abdalla EE Adam IJ Blair GE Boylston A Sue-Ling HM Finan P Johnston D The immunomodulatory effect of levamisole is influenced by postoperative changes and type of lymphocyte stimulant. Cancer Immunol Immunother. 1995; 41: 193–198. 7553689 10.1007/BF01521346 PMC11037643

[12] Macfarlane DG Bacon PA Levamisole-induced vasculitis due to circulating immune complexes. BMJ. 1978; 1: 407–408. 146534 10.1136/bmj.1.6110.407 PMC1602987

[13] Scheinberg MA Bezerra JB Almeida FA Silveira LA Cutaneous necrotising vasculitis induced by levamisole. BMJ. 1978; 1: 408. 10.1136/bmj.1.6110.408 PMC1602971 624030

[14] Rongioletti F Ghio L Ginevri F Bleidl D Rinaldi S Edefonti A Gambini C Rizzoni G Rebora A Purpura of the ears: a distinctive vasculopathy with circulating autoantibodies complicating long-term treatment with levamisole in children. Br J Dermatol. 1999; 140: 948–951. 10354040 10.1046/j.1365-2133.1999.02833.x

[15] Laux-End R Inaebnit D Gerber HA Bianchetti MG Vasculitis associated with levamisole and circulating autoantibodies. Arch Dis Child. 1996; 75: 355–356. 10.1136/adc.75.4.355-b PMC1511759 8984931

[16] Menni S Pistritto G Gianotti R Ghio L Edefonti A Ear lobe bilateral necrosis by levamisole-induced occlusive vasculitis in a pediatric patient. Pediatr Dermatol. 1997; 14: 477–479. 9436850 10.1111/j.1525-1470.1997.tb00695.x

[17] Barbano G Ginevri F Ghiggeri GM Gusmano R Disseminated autoimmune disease during levamisole treatment of nephrotic syndrome. Pediatr Nephrol. 1999; 13: 602–603. 10460511 10.1007/s004670050753

[18] Kopp SA High WA Green JJ Levamisole-induced Wegener’s granulomatosis following contaminated cocaine abuse. Skinmed. 2012; 10: 254–256. 23008947

[19] Weisner O Russell KA Lee AS Jenne DE Trimarchi M Gregorini G Specks U Antineutrophil cytoplasmic antibodies reacting with human neutrophil elastase as a diagnostic marker for cocaine-induced midline destructive lesions but not autoimmune vasculitis. Arthritis Rheum. 2004; 50: 2954–2965. 15457464 10.1002/art.20479

[20] Kouassi E Caillé G Léry L Larivière L Vézina M Novel assay and pharmacokinetics of levamisole and p-hydroxylevamisole in human plasma and urine. Biopharm Drug Dispos. 1986; 7: 71–89. 3754161 10.1002/bdd.2510070110

[21] Harper L Cockwell P Adu D Savage CO Neutrophil priming and apoptosis in anti-neutrophil cytoplasmic autoantibody-associated vasculitis. Kidney Int. 2001; 59: 1729–1738. 11318943 10.1046/j.1523-1755.2001.0590051729.x

[22] Trimarchi M Bussi M Sinico RA Meroni P Specks U Cocaine-induced midline destructive lesions - an autoimmune disease? Autoimmun Rev. 2013; 12: 496–500. 22940554 10.1016/j.autrev.2012.08.009

[23] Pearson T Bremmer M Cohen J Driscoll M Vasculopathy related to cocaine adulterated with levamisole: A review of the literature. Dermatol Online J. 2012; 18: 1. 22863623

[24] Pavenski K Vandenberghe H Jakubovic H Adam DN Garvey B Streutker CJ Plasmapheresis and steroid treatment of levamisole-induced vasculopathy and associated skin necrosis in crack/cocaine users. J Cutan Med Surg. 2013; 17: 123–128. 23582166 10.2310/7750.2012.12028

